# Genetically predicted causality between gut microbiota, blood metabolites, and intracerebral hemorrhage: a bidirectional Mendelian randomization study

**DOI:** 10.3389/fmicb.2024.1257405

**Published:** 2024-01-15

**Authors:** Tianlong Zhang, Gang Liu, Yina Cao, Jianqiang Zhao, Siyi Jiang, Ya Zhang, Min Li

**Affiliations:** ^1^Department of Critical Care Medicine, The Fourth Affiliated Hospital of Zhejiang University School of Medicine, Yiwu, Zhejiang, China; ^2^Department of Infection Control, The Fourth Affiliated Hospital of Zhejiang University School of Medicine, Yiwu, Zhejiang, China; ^3^Department of Neurology, The Fourth Affiliated Hospital of Zhejiang University School of Medicine, Yiwu, Zhejiang, China; ^4^Department of Cardiology, The Fourth Affiliated Hospital of Zhejiang University School of Medicine, Yiwu, Zhejiang, China; ^5^Department of Pharmacy, Yiwu Hospital of Traditional Chinese Medicine, Yiwu, Zhejiang, China

**Keywords:** gut microbiota, serum metabolites, intracerebral hemorrhage, genomewide association studies, Mendelian randomization

## Abstract

**Background:**

Recent research linked changes in the gut microbiota and serum metabolite concentrations to intracerebral hemorrhage (ICH). However, the potential causal relationship remained unclear. Therefore, the current study aims to estimate the effects of genetically predicted causality between gut microbiota, serum metabolites, and ICH.

**Methods:**

Summary data from genome-wide association studies (GWAS) of gut microbiota, serum metabolites, and ICH were obtained separately. Gut microbiota GWAS (*N* = 18,340) were acquired from the MiBioGen study, serum metabolites GWAS (*N* = 7,824) from the TwinsUK and KORA studies, and GWAS summary-level data for ICH from the FinnGen R9 (ICH, 3,749 cases; 339,914 controls). A two-sample Mendelian randomization (MR) study was conducted to explore the causal effects between gut microbiota, serum metabolites, and ICH. The random-effects inverse variance-weighted (IVW) MR analyses were performed as the primary results, together with a series of sensitivity analyses to assess the robustness of the results. Besides, a reverse MR was conducted to evaluate the possibility of reverse causation. To validate the relevant findings, we further selected data from the UK Biobank for analysis.

**Results:**

MR analysis results revealed a nominal association (*p* < 0.05) between 17 gut microbial taxa, 31 serum metabolites, and ICH. Among gut microbiota, the higher level of genus *Eubacterium xylanophilum* (odds ratio (OR): 1.327, 95% confidence interval (CI):1.154–1.526; Bonferroni-corrected *p* = 7.28 × 10^−5^) retained a strong causal relationship with a higher risk of ICH after the Bonferroni corrected test. Concurrently, the genus Senegalimassilia (OR: 0.843, 95% CI: 0.778–0.915; Bonferroni-corrected *p* = 4.10 × 10^−5^) was associated with lower ICH risk. Moreover, after Bonferroni correction, only two serum metabolites remained out of the initial 31 serum metabolites. One of the serum metabolites, Isovalerate (OR: 7.130, 95% CI: 2.648–19.199; Bonferroni-corrected *p* = 1.01 × 10^−4^) showed a very strong causal relationship with a higher risk of ICH, whereas the other metabolite was unidentified and excluded from further analysis. Various sensitivity analyses yielded similar results, with no heterogeneity or directional pleiotropy observed.

**Conclusion:**

This two-sample MR study revealed the significant influence of gut microbiota and serum metabolites on the risk of ICH. The specific bacterial taxa and metabolites engaged in ICH development were identified. Further research is required in the future to delve deeper into the mechanisms behind these findings.

## 1 Introduction

Spontaneous intracerebral hemorrhage (ICH) is one of the leading causes of morbidity and mortality worldwide (Li et al., 2017), posing a significant public health concern on a global scale. ICH, which is characterized by a high disability rate, currently lacks effective treatment options. Identifying modifiable risk factors for ICH that can be targeted for prevention strategies is crucial. Recently, there has been growing interest in the relationship between gut microbiota, human blood metabolites, and ICH. Changes in the composition of the gut microbiota influence the host immune system via inflammatory cytokine production and immune cell differentiation, thereby enhancing neuroinflammation in ICH (Luo et al., 2022). The gut microbiota produces a wide variety of metabolites, as well as other blood metabolites, which have systemic effects on humans. The specific serum metabolites are also believed to affect neuroinflammation in patients with ICH (Chen et al., 2022). ICH is characterized by the presence of bleeding within the brain parenchyma (de Oliveira Manoel et al., 2016), with subsequent neuroinflammation caused by the byproducts of blood metabolism within the same parenchyma (Alsbrook et al., 2023). Sustained activation of resident microglial cells, infiltration of systemic immune cells, and the generation of proinflammatory cytokines, chemokines, extracellular proteases, and reactive oxygen species are all hallmarks of neuroinflammation (Mracsko and Veltkamp, 2014). The release of these inflammatory mediators causes the breakdown of the blood–brain barrier, neuronal damage, and the onset of cerebral edema, ultimately exacerbating neurological dysfunction and resulting in a poor prognosis for the patient. Mitigating neuroinflammation is pivotal for enhancing patient outcomes; consequently, the gut microbiota and serum metabolites may play a significant role in ICH. Exploring the interplay between host genetics and the gut microbiota or serum metabolites is crucial for furthering our understanding of the ICH pathogenesis.

Although observational studies established links between gut microbiota, serum metabolites, and the risk of ICH development, residual confounding and reverse causality can influence these relationships. Clinical randomized trials represent the most effective way to test the findings of these studies. However, assessing the impact of gut microbiota, serum metabolites, and ICH is challenging due to cost and ethical concerns among the participants (Zabor et al., 2020).

Recently, MR has emerged as a popular alternative method for assessing the causal effects of related factors on diseases while avoiding biases stemming from confounding factors or reverse causality (Wu et al., 2020). MR analysis uses individual genetic variation, which is randomly distributed during conception, as an instrumental variable (Burgess et al., 2015). Instrumental variable data from extensive GWAS and identified SNPs related to the gut microbiota or serum metabolites are used to establish the causal relationship between exposures and outcomes.

Previous studies employed MR to analyze the gut microbiota and risk of sepsis (Chen et al., 2023), celiac disease (Li et al., 2023a). However, the relationship between gut microbiota, serum metabolites, and the risk of ICH has not been investigated in an MR study. Thus, the current study aims to estimate the effects of genetically predicted gut microbiota and serum metabolites on the risk of ICH. Consequently, several genetic variations associated with bacterial and metabolite composition that may drive ICH pathogenesis were identified. Our study findings can lay the groundwork for future research directions in ICH.

## 2 Materials and methods

### 2.1 Study design

**Figure 1:**
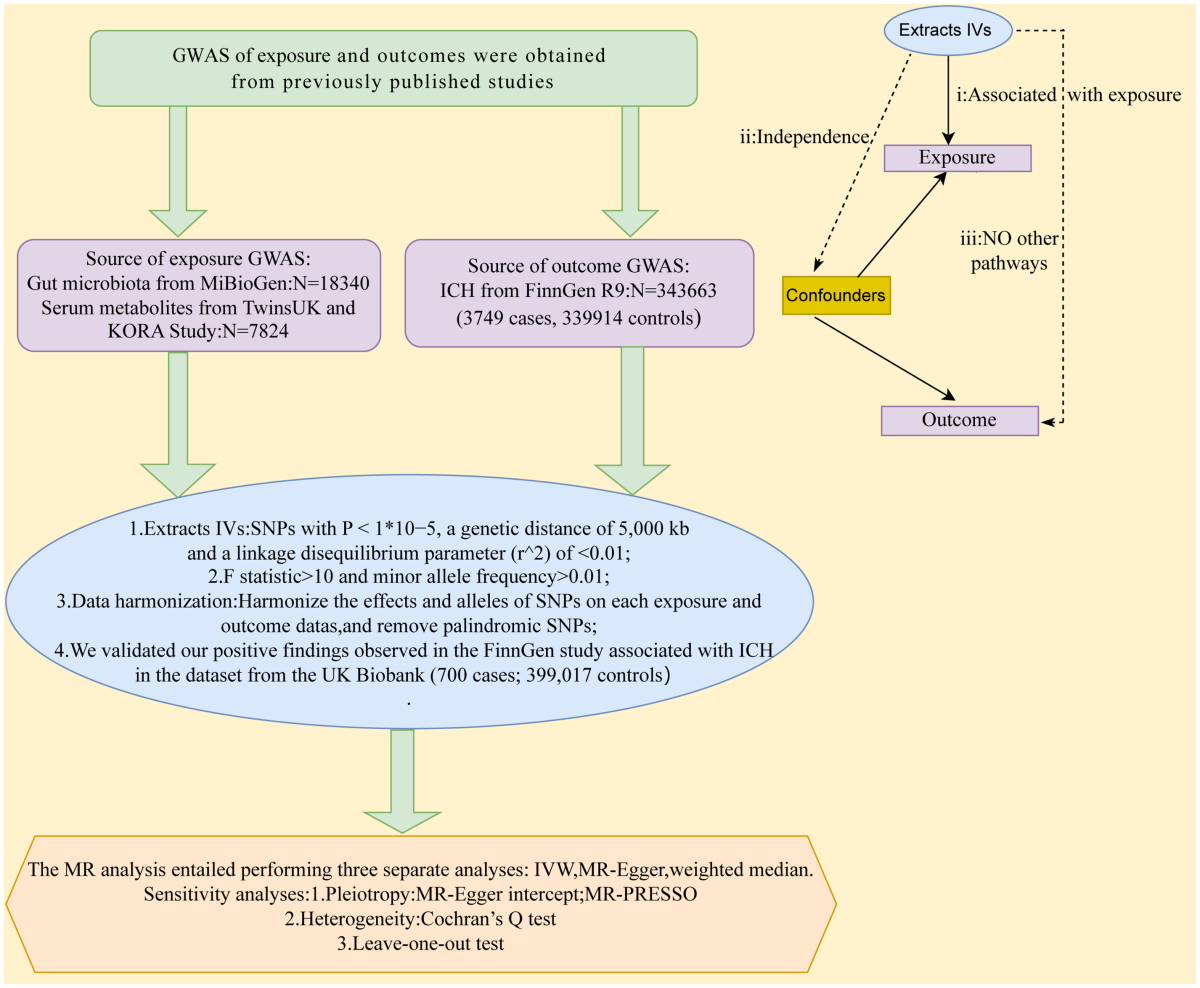
A schematic summary of the study design. GWAS, genome-wide association study; ICH, Intracerebral hemorrhage; IVs, inst1umental variable; SNPs, single-nucleotide polymorphisms; MR, Mendelian randomization; IVW, inverse-variance-weighted; MR-PRESSO, MR pleiotropy residual sum and outlier.

MR study was applied to investigate the causal effects between gut microbiota, serum metabolites, and ICH. Figure 1 depicts the schematic summary of the study design. The MR design should meet three necessary conditions (Figure 1): (i) The genetic variant selected as the instrumental variable (IV) should be associated with both gut microbiota and serum metabolites; (ii) the genetic instrument must be independent of potential confounding factors; (iii) the genetic variant has to be specifically associated with ICH through gut microbiota and serum metabolites, rather than through other pathways (Bowden and Holmes, 2019).

### 2.2 Data sources on the gut microbiota and serum metabolites

The comprehensive summary statistics of the genetic impact on the human gut microbiome in the MiBioGen Consortium are the most extensive collection of GWAS data on the gut microbiota to date, including genome-wide genotyping data from 18,340 individuals (14,363 of whom were of European descent) (Kurilshikov et al., 2021). In total, 211 taxa (16 classes, 35 families, 131 genera, 20 orders, and 9 phyla) were included in the microbiome quantitative trait loci mapping analysis. Summary-level data of GWAS for human metabolome were generated from the TwinsUK and KORA studies, which involved 7,824 participants. In the GWAS, 486 metabolite concentrations were tested (Shin et al., 2014). In the current study, we excluded four bacterial traits for which IVs could not be extracted, resulting in a remaining set of 207 bacterial traits (16 classes, 34 families, 128 genera, 20 orders, and 9 phyla) for further analysis. Similarly, 451 serum metabolites were utilized for the subsequent analysis.

### 2.3 Instrumental variables selection

Single-nucleotide polymorphisms (SNPs) were selected with *p*-values below the locus-wide significance level (1 × 10^−5^) as instrumental variables in the preliminary analysis to obtain more comprehensive results and increase sensitivity to IVs. Next, all IVs underwent linkage disequilibrium (LD) clumping (*r*^2^ < 0.01; distance = 5,000 kb) to reduce the influence of correlations between SNPs. Moreover, PhenoScanner^1^ was used to search for genome-wide traits that were significantly associated with these SNPs to assess confounding factors that potentially associated with gut microbiota, serum metabolites, and ICH (Flatby et al., 2023). Furthermore, the F-statistic (*R*^2^ (*N*–2)/(1–*R*^2^)) was calculated to evaluate the strength of each instrument, where *R*^2^ is equal to the proportion of variance explained by the genetic instrument, and N is the effective sample size of the GWAS. Notably, an F-statistic threshold greater than 10 indicates that the genetic variation has a relatively robust estimation effect in MR analysis (Burgess and Thompson, 2011). SNPs with an F-statistic lower than 10 and palindromic SNPs (where it was unclear which allele was the effect allele) (Xu et al., 2021) were excluded from this study. Ultimately, 2,249 host SNPs (*p* < 1 × 10^−5^) associated with gut microbiota and 8,595 host SNPs (*p* < 1 × 10^−5^) associated with serum metabolites as instrumental variables were identified. Supplementary Tables S1, S2 provide detailed information on the selected genetic variants.

### 2.4 Data sources on the ICH

Summary-level data of GWAS for ICH were generated from the latest FinnGen R9 biobank (Kurki et al., 2023), which included 3,749 ICH cases and 339,914 controls. More details on the ICH GWAS can be found online.^2^

### 2.5 MR analysis

In the current study, a two-sample MR analysis was used to assess the causal association between gut microbiota, serum metabolites, and ICH. The method entailed performing three separate analyses: the random-effects inverse variance-weighted (IVW) method, weighted median estimate, and MR-Egger regression estimate.

The random-effects IVW method was used as the primary analysis for MR. It provides unbiased estimates by considering the potential presence of horizontal pleiotropy or achieving balance in horizontal pleiotropy (Burgess et al., 2013). Concurrently, the MR-Egger and weighted median were employed as sensitivity analysis methods. Sensitivity analysis methods were used to consider directional horizontal pleiotropy, which refers to the possibility of SNP effects on target outcomes through other biological pathways independent of the exposure under investigation (Verbanck et al., 2018). The MR-Egger method accommodates directional horizontal pleiotropic effects. If the intercept term differs from zero, then not all included instruments are effective, and the IVW may be biased (Bowden et al., 2015). If at least half of the instruments are valid, a weighted median approach is used to calculate causal estimates for each SNP. Then, the resulting overall MR estimate is determined by taking the median of these estimates (Bowden et al., 2016). The Mendelian Randomization Pleiotropy RESidual Sum and Outlier (MR-PRESSO) test was also performed to identify possible horizontal pleiotropy and correct for its influence by removing outliers (Verbanck et al., 2018). Leave-one-out analyses were conducted to assess further the pleiotropy associated with individual SNPs. In addition, potential heterogeneity and outliers were examined using *I*^2^ and Cochran Q-derived *p* values.

A value of *p* < 0.05 was considered as nominal association. The Bonferroni-corrected test was utilized (Brion et al., 2013), considering the number of bacteria in each attribute group, to establish a more robust causal association (classes: 0.05/16 (3.13 × 10^−3^), families: 0.05/34 (1.47× 10^−3^), genera: 0.05/128 (3.90 × 10^−4^), orders: 0.05/20 (2.50 × 10^−3^), and phyla: 0.05/9 (5.56 × 10^−3^)). The Bonferroni-corrected *p*-value for serum metabolites was 0.05/451 (1.11 × 10^−4^). The statistical analyses were performed using R software version 4.2.3. MR analyses were conducted using the TwoSampleMR package (Zhou et al., 2019).

### 2.6 Reverse MR analysis

A reverse MR analysis was conducted to assess whether ICH has any causal effects on gut microbiota abundance or serum metabolites. MR analysis was performed as described earlier.

### 2.7 Gut microbiota and serum metabolites bidirectional MR analysis

An additional bidirectional MR analysis was performed to determine if significant gut microbiota had any reciprocal causal effects on significant serum metabolites. We used the genus *Eubacterium xylanophilum* or the genus Senegalimassilia as the exposure and Isovalerate as the outcome. Similarly, we used Isovalerate as the exposure and the genus *Eubacterium xylanophilum* or genus Senegalimassilia as the outcome.

### 2.8 Validation set

We utilized publicly available summary statistics data on ICH (700 cases, 399,017 controls) from an independent European ancestry cohort sourced from the UK Biobank (Gagliano Taliun et al., 2020) to validate our positive findings observed in the FinnGen study.

## 3 Results

### 3.1 Causal effects of gut microbiota on ICH

**Figure 2:**
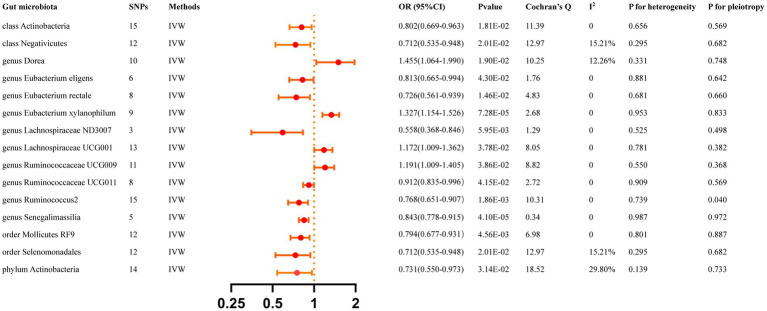
Causal effects of gut microbiota 0n ICH. Significant *p*-value after Bonferroni-corrected classes: 0.05/16 (3.13 × 10–3), families: 0.05/34 (l.47 > < 10–3), genera: 005/128 (3.90 × 10–4), orders: 0.05/20 (2.50 × 10–3), and phyla: 0.05/9 (5.56 × 10–3).

All reported associations correspond to an OR for risk of ICH per standard deviation (SD) change in abundance of gut microbiota feature. The IVW method identified 17 causal associations from gut microbiota features to ICH traits were identified (Supplementary Table S3). Due to two unknown bacterial taxa, we excluded them from the presentation of the results. Figure 2 presents these IVW results (*p* < 0.05).

The IVW analyses demonstrated that genetically greater abundance of class Actinobacteria (OR: 0.802, 95% CI: 0.668–0.963, *p* = 0.018), class Negativicutes (OR: 0.711, 95% CI: 0.535–0.948, *p* = 0.020), genus *Eubacterium eligens* (OR: 0.813, 95% CI: 0.665–0.994, *p* = 0.043), genus *Eubacterium rectale* (OR: 0.726, 95%CI: 0.561–0.939, *p* = 0.015), genus Lachnospiraceae ND3007 (OR: 0.558, 95% CI: 0.368–0.846, *p* = 0.006), genus Ruminococcaceae UCG011 (OR: 0.912, 95% CI: 0.835–0.996, *p* = 0.042), genus Ruminococcus2 (OR:0.768, 95%CI:0.651–0.907, *p* = 0.002), genus Senegalimassilia (OR: 0.843, 95% CI: 0.778–0.915, *p* = 4.10 × 10^−5^), order Mollicutes RF9 (OR: 0.794, 95% CI: 0.677–0.931, *p* = 0.005), order Selenomonadales (OR: 0.712, 95% CI: 0.535–0.948, *p* = 0.020), and phylum Actinobacteria (OR: 0.731, 95% CI: 0.550–0.973, *p* = 0.031) were correlated with a reduced risk of ICH. Additionally, the genetically predicted abundance of genus Dorea (OR: 1.455, 95% CI: 1.064–1.990, *p* = 0.019), genus *Eubacterium xylanophilum* (OR: 1.327, 95% CI:1.154–1.526, *p* = 7.28 × 10^−5^), genus Lachnospiraceae UCG001 (OR: 1.172, 95% CI: 1.009–1.362, *p* = 0.038), and genus Ruminococcaceae UCG009 (OR: 1.191, 95% CI: 1.009–1.405, *p* = 0.039) were positively correlated with the risk of ICH.

The MR-Egger regression found no evidence of horizontal pleiotropy, as indicated by the lack of significance for all intercepts (*p* > 0.05). Genus *Eubacterium rectale* was identified as an outlier by MR-PRESSO, and the outlier was subsequently corrected. No remaining results were observed as outliers by MR-PRESSO, indicating the absence of significant directional horizontal pleiotropy. The results of Cochran’s Q for the IVW test did not detect evidence of heterogeneity (Supplementary Table S5). Supplementary Table S10 displays the genetic associations between SNPs related to significant gut microbiota and their respective outcomes, as identified through PhenoScanner. Additional File S1 presents the leave-one-out analysis.

### 3.2 Causal effects of serum metabolites on ICH

**Figure 3:**
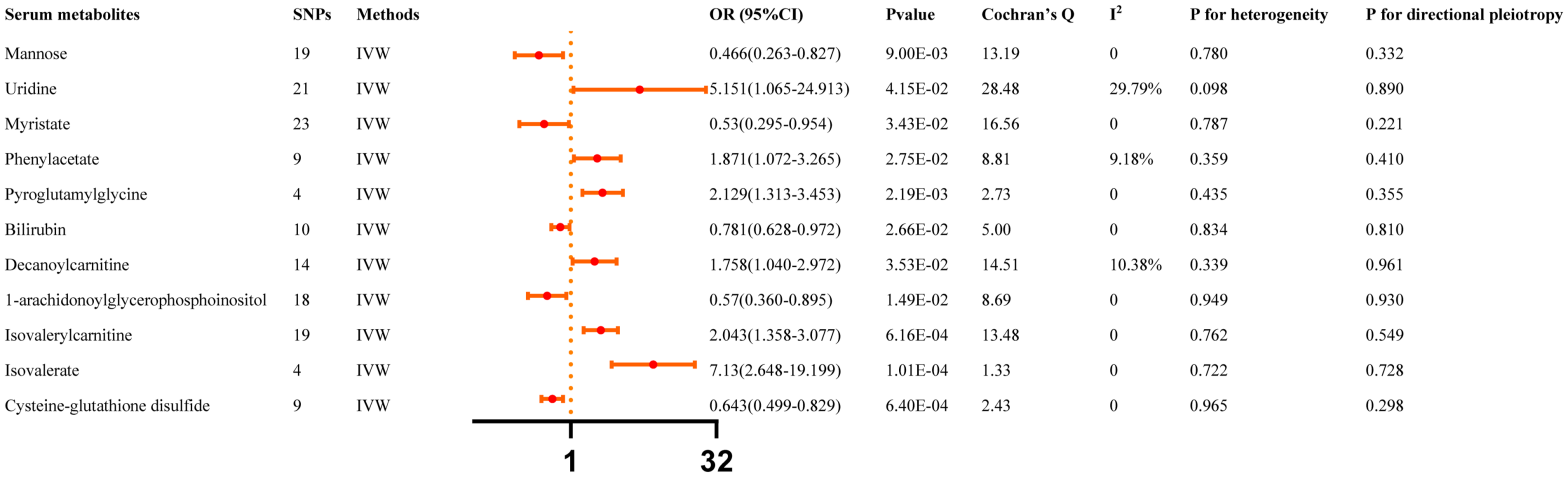
Causal effects of Serum metabolites on ICH. The Bonferroni-corrected *p*-value for serum metabolites was 005/451 (1.11 × 10–4).

All reported associations correspond to an OR for risk of ICH per SD change in the abundance of gut microbiota features. The IVW method revealed 31 causal relationships between serum metabolites and ICH traits (Supplementary Table S4). However, 20 of these metabolites were unknown and were excluded from the presentation of the results. Figure 3 presents these IVW results (*p* < 0.05).

IVW results indicated that genetically predicted higher concentrations of Mannose (OR: 0.466, 95% CI: 0.263–0.827, *p* = 0.009), Myristate (OR: 0.530, 95% CI: 0.295–0.954, *p* = 0.034), Bilirubin (OR: 0.781, 95% CI: 0.628–0.972, *p* = 0.027), 1-arachidonoylglycerophosphoinositol (OR: 0.570, 95% CI: 0.360–0.895, *p* = 0.015), Cysteine-glutathione disulfide (OR: 0.643, 95% CI: 0.499–0.829, *p* = 6.40 × 10^−4^) were correlated with a reduced risk of ICH. Moreover, genetically predicted higher concentrations of Uridine (OR: 5.151, 95% CI: 1.065–24.913, *p* = 0.042), Phenylacetate (OR: 1.871, 95% CI: 1.072–3.265, *p* = 0.027), Pyroglutamylglycine (OR: 2.129, 95% CI: 1.313–3.453, *p* = 0.002), Decanoylcarnitine (OR: 1.758, 95% CI: 1.040–2.972, *p* = 0.035), Isovalerylcarnitine (OR: 2.043, 95% CI: 1.358–3.077, *p* = 6.16 × 10^−4^), and Isovalerate (OR: 7.130, 95% CI: 2.648–19.199, *p* = 1.01 × 10^−4^) were associated with higher ICH risk. The MR-Egger regression did not find evidence of horizontal pleiotropy and Cochran’s Q test did not detect evidence of heterogeneity. Decanoylcarnitine was identified as an outlier by MR-PRESSO and the outlier was subsequently corrected. No remaining results were observed as outliers by MR-PRESSO, indicating no significant directional horizontal pleiotropy (Supplementary Table S6). Finally, Additional File S1 depicts the leave-one-out analysis.

### 3.3 Reverse MR analysis

#### 3.3.1 Modification of gut microbiota by ICH

**Figure 4:**
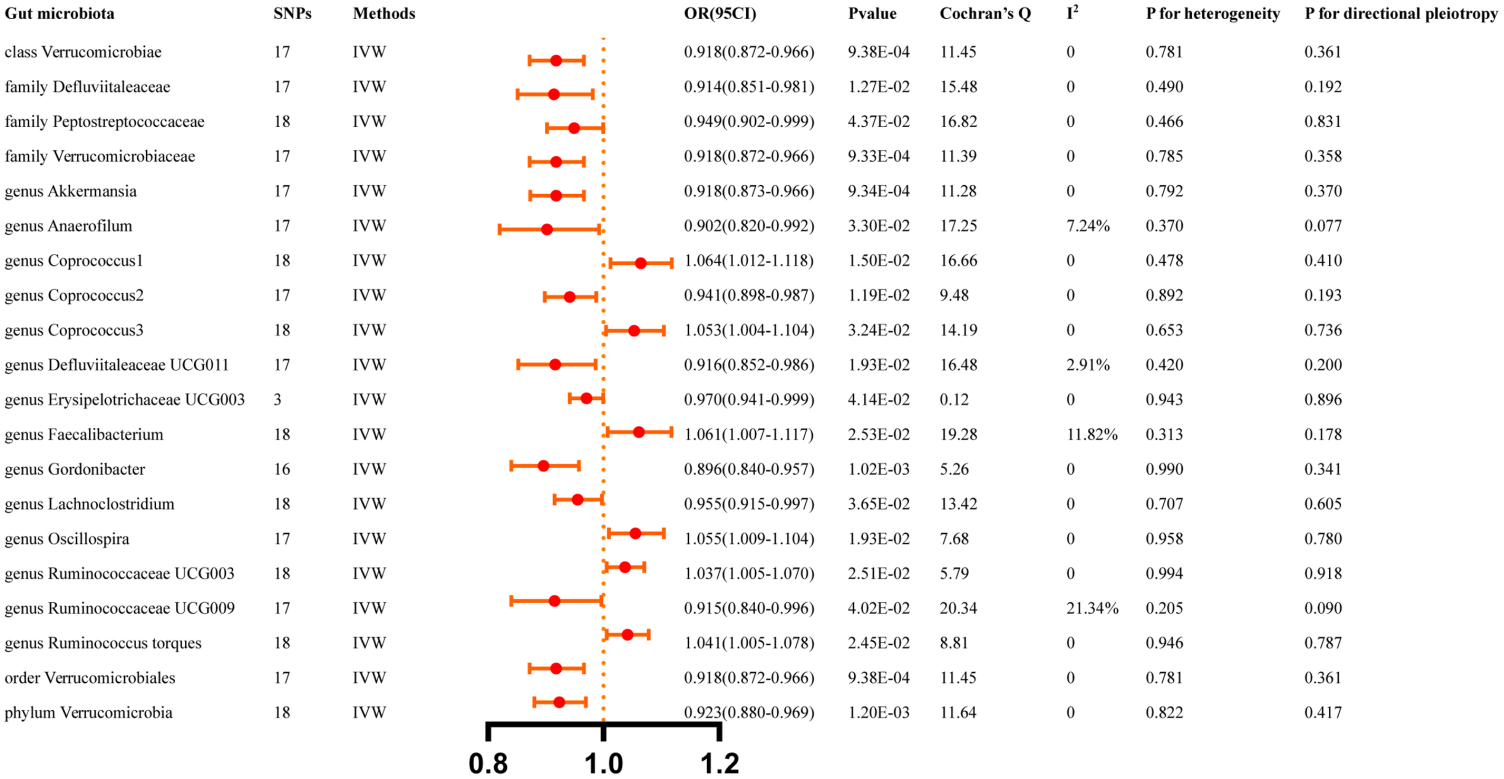
Causal effects of ICH on gut microbiota. Significant *p-*value after Bonferroni-corrected classes: 0.05/16 (3.13 × 10–3), families: 0.05/34 (l.47 > < 10–3), genera: 005/128 (3.90 × 10–4), orders: 0.05/20 (2.50 × 10–3), and phyla: 0.05/9 (5.56 × 10–3).

Figure 4 summarizes the causal relationship between ICH and bacterial taxa. After Bonferroni correction, ICH was causally and significantly associated with a lower abundance of class Verrucomicrobiae (OR: 0.918, 95%CI: 0.872–0.966, *p* = 9.38 × 10^−4^), family Verrucomicrobiaceae (OR: 0.918, 95% CI: 0.872–0.966, *p* = 9.33 × 10^−4^), order Verrucomicrobiales (OR: 0.918, 95% CI: 0.872–0.966, p = 9.38 × 10^−4^), and phylum Verrucomicrobia (OR: 0.923, 95% CI: 0.880–0.969, *p* = 1.20 × 10^−3^). Supplementary Table S6 lists the details and the other sensitivity methods.

#### 3.3.2 Modification of serum metabolites by ICH

**Figure 5:**
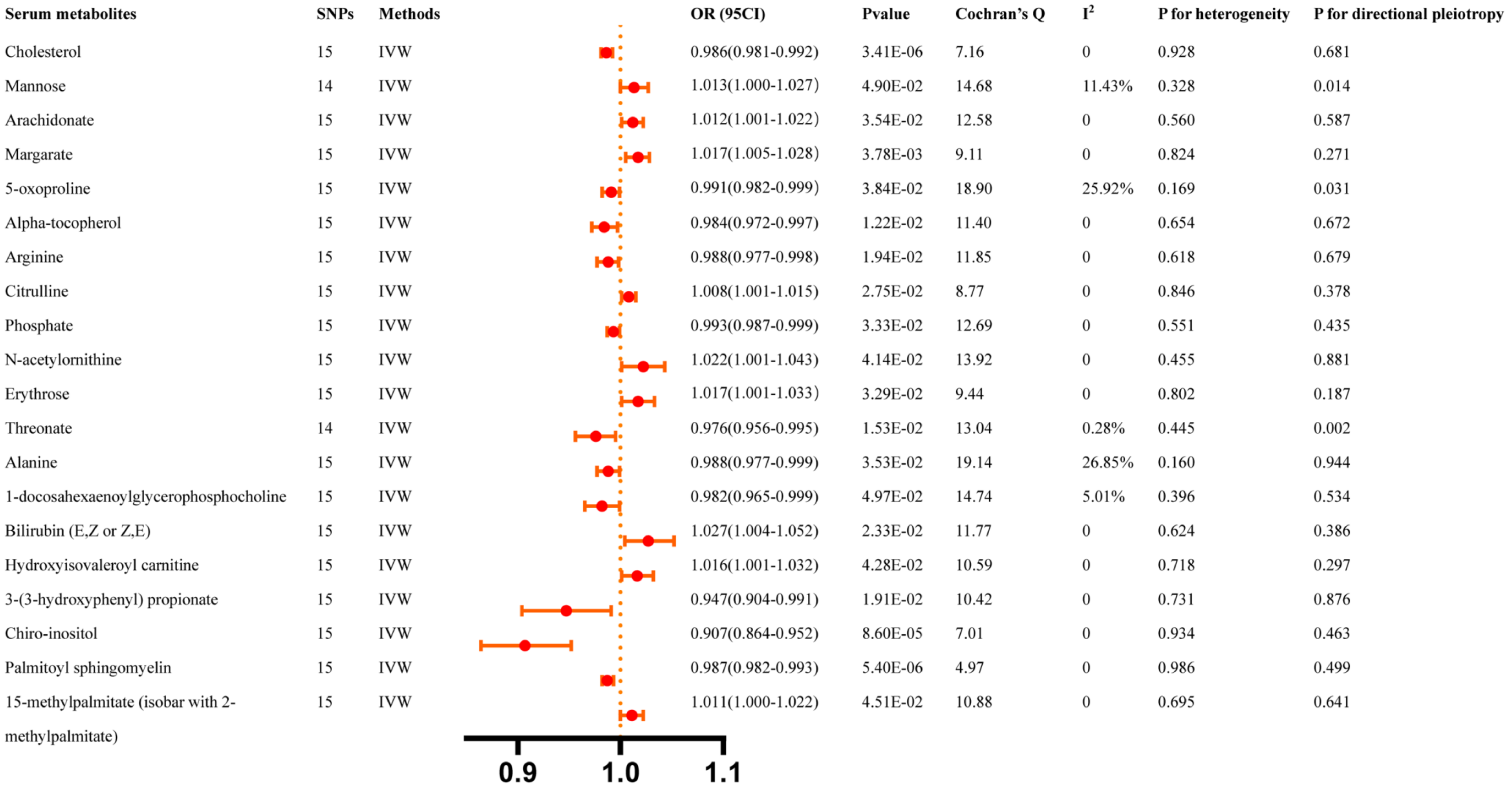
Casual effects of ICH on serum metabolites. The Bonferroni-corrected *p*-value for serum metabolites was 005/451 (1.11 × 10).

Figure 5 summarizes the causal relationship between ICH and serum metabolites. After Bonferroni correction, ICH was causally and significantly associated with lower concentrations of Cholesterol (OR: 0.986, 95% CI: 0.981–0.992, *p* = 3.41 × 10^−6^), Chiro-inositol (OR: 0.907, 95% CI: 0.864–0.952, *p* = 8.6 × 10^−5^), and Palmitoyl sphingomyelin (OR: 0.987, 95% CI: 0.982–0.993, *p* = 5.4 × 10^−6^). Supplementary Table S7 lists the details and the other sensitivity methods.

### 3.4 Gut microbiota and serum metabolites bidirectional MR analysis

A bidirectional MR analysis was performed again to evaluate the potential causal relationship between significant gut microbiota and significant serum metabolites. The results of the MR analysis reveal no causal relationship between the genus *Eubacterium xylanophilum* (*p* = 0.623), genus Senegalimassilia (*p* = 0.914), and isovalerate. Moreover, there is no causal association between isovalerate and the genus *Eubacterium xylanophilum* (*p* = 0.560) or genus Senegalimassilia (*p* = 0.869) (Supplementary Table S9). The analysis findings provide supporting evidence for our conclusion that both gut microbiota and serum metabolites have distinct influences on the incidence of ICH.

### 3.5 Validation set

We validated the causal relationships of 17 gut microbiota and 31 serum metabolites associated with ICH in the dataset from the UK Biobank. The results indicate that the presence of the following genera is associated with intracerebral hemorrhage (ICH): *Eubacterium eligens* (OR: 0.474, 95% CI: 0.246–0.913, *p* = 0.026), Ruminococcaceae UCG011 (OR: 0.697, 95% CI: 0.550–0.884, *p* = 2.84 × 10^−3^), Ruminococcus2 (OR: 0.605, 95% CI: 0.381–0.962, *p* = 0.034), and Dorea (OR: 1.919, 95% CI: 1.119–3.291, *p* = 0.018). Additionally, among the metabolites, only one unknown metabolite was found to have a causal association, and it was excluded from our analysis (Supplementary Tables S11, S12).

## 4 Discussion

**Figure 6:**
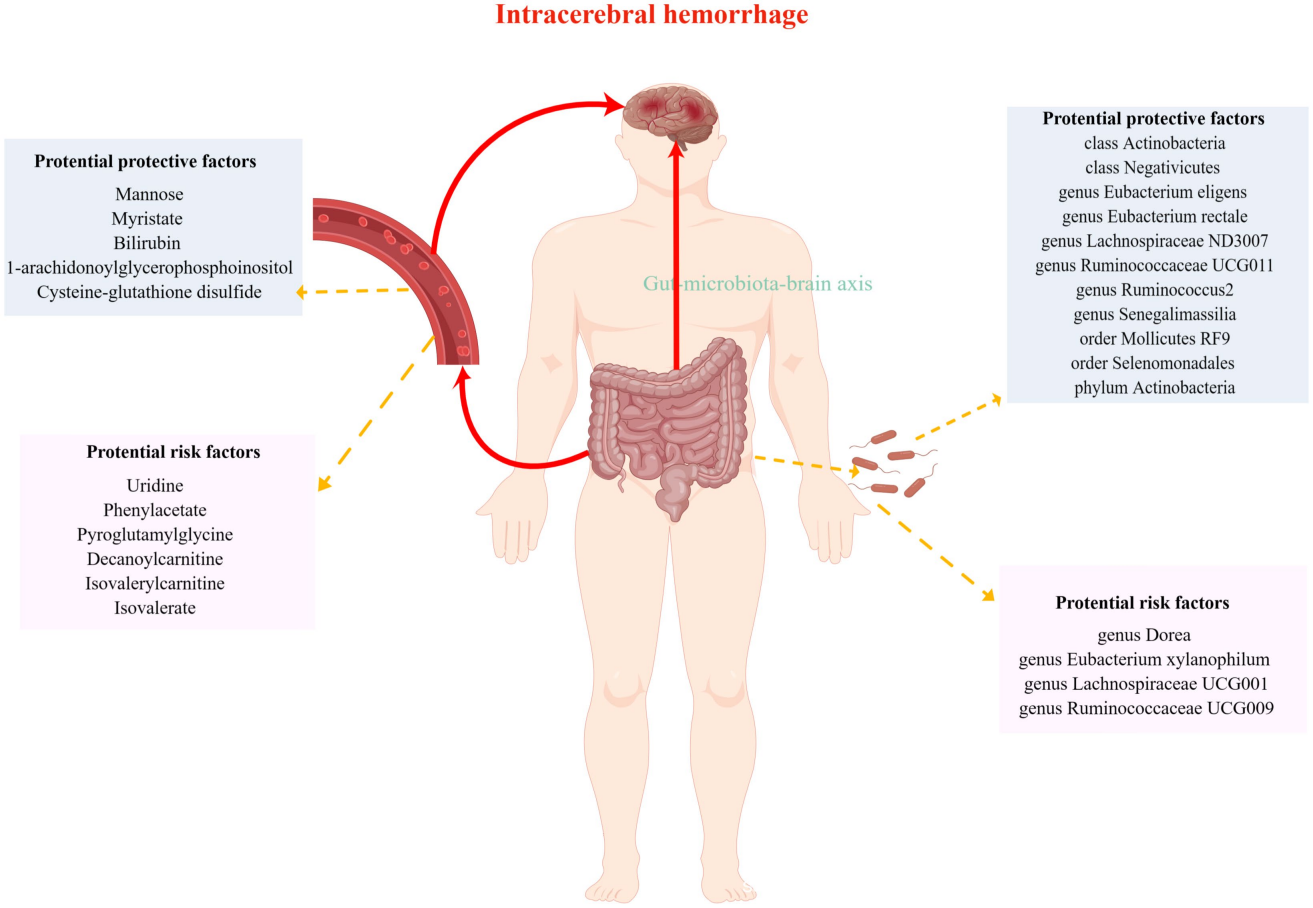
Casual links between gut microbiota, serum metabolites and intracerebral hemorrhage.

Over the last few decades, rapid advances in microbiome and metabolome studies have greatly aided our understanding of the pathogenic mechanisms underlying ICH (Zhang et al., 2021b; Chen et al., 2022). Most studies are animal or case–control studies, which can demonstrate an association with ICH but cannot establish a causal relationship. To the best of our knowledge, this is the first MR analysis report establishing a causal relationship between gut microbiota/serum metabolites and ICH. In our MR study, we excluded unknown bacteria or metabolites and found associations between 15 gut microbiota taxa and 11 serum metabolites with ICH (Figure 6). After performing Bonferroni correction, the genus *Eubacterium xylanophilum* and Isovalerate were found to have a strong causal relationship with a higher risk of ICH, as well as the genus Senegalimassilia with a strong causal relationship with a lower risk of ICH. Sensitivity analyses further supported this conclusion.

However, in the validation set, following Bonferroni correction (0.05/17), only the genus Ruminococcaceae UCG011 remains significantly associated with a reduced risk of ICH. Discrepancies in the results between the two datasets may be attributed to a relatively lower number of intracerebral hemorrhage cases in the UK Biobank and incomplete updates in the database. The FinnGen R9 Biobank, being the most recent and having the largest number of ICH cases, provides potentially more comprehensive results. Consequently, the subsequent discussion primarily focuses on the relevant findings from the FinnGen database.

The relationship between these florae in our findings and ICH has rarely been reported. Previous studies demonstrated significant differences in the overall abundance of gut microbes, as well as specific flora, in patients with ICH compared to healthy control individuals. A prior case–control study revealed that an enriched presence of Enterococcus and a depleted presence of Prevotella increased the risk of ICH (Luo et al., 2022). Another prospective case–control study depicted that Escherichia/Shigella, Enterococcus Peptoniphilus, and Ezakiella, are more abundant in the bacterial flora subsequent to ICH (Haak et al., 2021). Multi-omics research revealed a significant decrease in the phylum of Firmicutes and a significant increase in Bacteroidetes in ICH patients. At the genus level, Streptococcus, Lactobacillus, Bifidobacterium, and Akkermansia were found to be abundant in ICH patients (Chen et al., 2022). Moreover, Li et al. (2022b) and Xiong et al. (2022) reported that the abundance of Akkermansia in patients with parenchymal hemorrhage was significantly increased. Several animal model studies consistently indicated that ICH is characterized by a significant increase in Actinomycetes, Bacteroidetes, and Firmicutes, with minor changes in other bacterial populations (Yu et al., 2021a; Zhang et al., 2021a). The gut microbiota affects the occurrence of ICH, and similarly, patients with ICH also exhibit alterations in their gut microbiota. Our study presents divergent findings, revealing that an enriched abundance of the genus *Eubacterium xylanophilum* increases the risk of ICH. In contrast, an enriched abundance of the genus Senegalimassilia decreases the risk of ICH. Among the 13 meaningful results that we excluded, some findings support the results of the aforementioned study. However, potentially meaningful results may have been lost due to the overly stringent nature of the Bonferroni correction. These findings are not currently discussed in this article, but they can provide directions for future research. The genus *Eubacterium xylanophilum* is considered a pathogenic bacterium in the gut and was found to be associated with diseases such as colitis in mice (Li et al., 2022a) and upper urinary urolithiasis (Zhang et al., 2023). *Senegalimassilia* is a genus of actinobacterial strains that are Gram-positive, anaerobic, and coccobacilli-shaped (Han et al., 2020). The genus *Senegalimassilia* was found to be a protective factor for various diseases, including hypertension (Li et al., 2023b), allergic conjunctivitis (Liu et al., 2023). Based on our findings, this could potentially provide a strategy for extracting this bacterium from fecal samples in the future to prevent a specific disease. Although the microbiota’s heterogeneity and interindividual differences diminish the statistical power of microbiota analysis, it still offers a favorable basis for exploring the causal relationship between bacterial taxa and ICH.

Emerging research suggested that the gut microbiome can influence various physiological processes, including neurological functions, metabolism, and immune responses (Yu et al., 2022). Regarding neural function, the gut microbiota may form a direct functional link with the central nervous system (CNS) through the gut-brain axis (Berer et al., 2011). The gut microbiota plays a key role in CNS diseases, such as ischemic stroke (Chen et al., 2019), and ICH (Yu et al., 2021b), which may be related to neuroinflammation. The potential mechanism by which the gut microbiota influences the risk of ICH may include neuroinflammation modulation through its impact on T-cell homeostasis. When there is an imbalance in the gut microbiota, inflammatory factors in the gut, such as T helper type 1, Th17, and interleukin-6, are released in significant amounts. The release of these inflammatory factors causes alterations in intestinal permeability, barrier dysfunction, and the extravasation of inflammatory substances into the peripheral blood, which are then transported to the blood–brain barrier. Finally, these factors influence the occurrence, progression, and prognosis of ICH within the cerebrovascular system (Zou et al., 2022). Furthermore, the gut microbiota functions like an endocrine organ that produces bioactive metabolites, including short-chain fatty acids, neurotransmitter precursors, secondary bile acids, trimethylamine-N-oxide, and lipopolysaccharide (Xu et al., 2020). Gut-derived metabolites, along with other blood metabolites in the human body, play a part in the onset and development of diseases. While the exact mechanisms are unknown, these metabolites have the potential to impact ICH outcomes by modulating neuroinflammation, influencing brain function, and possibly contributing to cardiovascular factors (Wang and Zhao, 2018). The gut-brain connection in ICH is an evolving area of research, and further studies are warranted to understand its implications fully.

Regarding serum metabolites, the association between these metabolites in our findings and ICH has rarely been reported. Previous studies demonstrated the crucial role of blood metabolites in the early diagnosis and prognosis of patients with cerebral hemorrhage. A prior metabolomic analysis of patients with ICH revealed that the 20-hydroxy-leukotriene B4 metabolite may function as a potential biomarker for ICH diagnosis and risk assessment (Zhang et al., 2021b). Another metabolomic study aimed at distinguishing between cerebral hemorrhage and acute ischemic stroke confirmed the potential roles of 11 metabolites (Zhang et al., 2017). However, different outcomes have been reported. A case–control study demonstrated that the 225 metabolic markers showed no concordant associations with ICH (Holmes et al., 2018). Our research findings indicated that 11 serum metabolites are associated with ICH. However, after Bonferroni correction, only isovalerate remains strongly correlated with ICH, and an elevated level of isovalerate increases the risk of ICH by more than sevenfold. Isovalerate is a short-chain fatty acid produced by gut microbiota (Fawad et al., 2022). Previous studies revealed that there is an association between isovalerate and adenomatous polyposis (Niccolai et al., 2019). However, no clinical or experimental studies supported the claim that ICH affects blood isovalerate levels. Based on our findings, this opens up the possibility for future research to investigate the use of this biomarker in blood assays for the prevention of ICH. However, additional studies are required to validate its efficacy.

The relationship between gut microbiota, serum metabolites, and ICH has remained controversial due to the possibility of confounding risk factors in observational studies. Thus, in this research, an MR study was conducted to investigate this association in order to address this issue. Besides, a reverse MR analysis was performed to account for reverse causality. The reverse MR analysis produced significant results that contradicted our main findings, bolstering our conclusions. Additionally, this suggested that while gut microbiota and metabolites influence ICH, ICH also exerts an impact on gut microbiota and metabolites. Moreover, to enhance the reliability of our findings, the causal relationships between significant gut microbiota and serum metabolites were assessed. The results revealed no causal relationship between them, suggesting that they independently contribute to ICH. In a sense, this research provides a theoretical foundation for follow-up research.

Nevertheless, this study has several limitations. First, the GWAS data of exposure (gut microbiota and metabolites) and outcome (ICH) were obtained from publicly available summary data, and the potential overlap in samples introduces a challenging risk of confounding bias (Burgess et al., 2019). Second, the study participants were predominantly individuals of European descent. Considering that ICH is a global public health issue, it is important to evaluate the generalizability of our findings to other populations. Third, to enhance the reliability of our results, multiple corrected analyses were employed. However, the stringent multiple testing correction may overlook potential bacterial strains or metabolites causally linked to ICH. Fourth, we did not conduct further stratified analyses on the data, such as age or gender stratification among ICH patients, to investigate potential differences. Finally, due to the limited variance explained by SNPs or sample size limitations in GWAS results, some of our MR analyses may have lacked sufficient power to detect small effects. Despite these potential limitations, this study provides the best available evidence on the causal impacts of gut microbiota and serum metabolites on the risk of ICH. Prospective studies to investigate the underlying mechanisms of treatment or prevention strategies are required to develop effective and feasible treatment or prevention strategies.

## 5 Conclusion

This two-sample MR study revealed the significant role of gut microbiota and serum metabolites in the risk of ICH. The identification of the genus *Eubacterium xylanophilum*, genus Senegalimassilia, and isovalerate as strong causal factors, as well as the nominal causality of other microbiota or metabolites, adds to our understanding of the complex interplay between the gut, metabolites, and the brain in ICH development. Furthermore, they offer valuable insights and strategies for ICH prevention and treatment. Further research is warranted to delve deeper into the mechanisms underlying these findings.

## Data availability statement

The datasets presented in this study can be found in online repositories. The names of the repository/repositories and accession number(s) can be found in the article/Supplementary material.

## Author contributions

TZ: Data curation, Writing – original draft. GL: Data curation, Writing – original draft. YC: Writing – original draft. JZ: Data curation, Writing – original draft. SJ: Visualization, Writing – original draft. YZ: Visualization, Writing – original draft. ML: Writing – review & editing.

## Footnotes

^1^ http://www.phenoscanner.medschl.cam.ac.uk/

^2^ https://www.finngen.fi/en
